# Personal health monitoring in the armed forces – scouting the ethical dimension: A case study in the Netherlands Armed Forces during the Covid-19 pandemic

**DOI:** 10.1186/s12910-023-00899-9

**Published:** 2023-03-09

**Authors:** Dave Bovens, Eva van Baarle, Bert Molewijk

**Affiliations:** 1 Department of Ethics, Law and Humanities, Amsterdam UMC, location VU University Medical Center, De Boelelaan 1089a, 1081HV, Amsterdam, The Netherlands; 2 Defence Healthcare Organisation, Ministry of Defence, Utrecht, The Netherlands; 3 Faculty of Military Sciences, Netherlands Defence Academy, Breda, The Netherlands

**Keywords:** Personal health monitoring, Armed forces, Values, Moral dilemmas, Responsible, Ethical dimension, Ethics support

## Abstract

**Background:**

The field of personal health monitoring (PHM) develops rapidly in different contexts, including the armed forces. Understanding the ethical dimension of this type of monitoring is key to a morally responsible development, implementation and usage of PHM within the armed forces. Research on the ethics of PHM has primarily been carried out in civilian settings, while the ethical dimension of PHM in the armed forces remains understudied. Yet, PHM of military personnel by design takes place in a different setting than PHM of civilians, because of their tasks and the context in which they operate. This case study therefore focusses on obtaining insights into the experiences and related values of different stakeholders regarding an existing form of PHM, the Covid-19 Radar app, in the Netherlands Armed Forces.

**Methods:**

We carried out an exploratory qualitative study, using semi-structured interviews with twelve stakeholders in the Netherlands Armed Forces. We focussed on participation in the use of PHM, reflections on the practical use and use of data, moral dilemmas and the need for ethics support, all in regard to PHM. The data was analysed using an inductive thematic approach.

**Results:**

Three interlinking categories reflecting ethical dimensions of PHM emerged: (1) values, (2) moral dilemmas, and (3) external norms. The main values identified were: security (in relation to data), trust and hierarchy. Multiple related values were found. Some, but no broadly shared, moral dilemmas were identified and no strong need for ethics support was expressed.

**Conclusion:**

This study shed light on key values, provide insights in the experienced and presumed moral dilemmas and bring to mind ethics support considerations when looking at PHM in the armed forces. Some values bring a certain vulnerability to military users when personal and organisational interests are not aligned. Furthermore, some identified values may hinder a careful consideration of PHM because they potentially conceal parts of ethical dimensions of PHM. Ethics support can assist in uncovering and addressing these concealed parts. The findings highlight a moral responsibility for the armed forces to devote attention to the ethical dimensions of PHM.

## Background

Look at your wrist: many of you probably wear a smartwatch to assess health-related parameters, with the aim to improve your health and longevity. The field of personal health monitoring (PHM) develops rapidly. Measuring blood glucose with a sensor and smartphone, sleep tracking, fall risk assessment and gait analysis, continuous ECG, activity tracking, all are examples of more and more common forms of PHM. The global mobile health market is expected to grow by 11% annually, from an estimated USD 56.8 billion in 2022 to USD 130.6 billion in 2030 [1]. An aging population with more chronic illnesses, the increasing costs of healthcare, a shift to personalized medicine and the internet of things are all relevant contributors to the rising demand and possibilities of PHM [1–4].

PHM can be defined as “any electronic device or system that longitudinally monitors and records data about a health-related aspect of a person’s life” [5]. PHM can be used in civilian (public) healthcare for a wide variety of tasks, such as prevention (e.g. remote monitoring for congestive heart failure [6]), treatment (e.g. continuous blood glucose monitoring in diabetes), support (e.g. mobile Covid-19 contact tracing [7]) and rehabilitation (e.g. limp rehabilitation for stroke survivors [8]). Besides the use of PHM in healthcare, it is also recreationally used by an increasing amount of people, e.g. to keep track of and to assist in physical activities. Furthermore, PHM is emerging in occupational health management, for example in the health promotion of employees [9]. PHM offers various opportunities and matching benefits like data monitoring and analysis, pattern recognition and diagnosis, lifestyle advice and early warning in medical emergencies [5].

PHM is also researched in the context of the armed forces, since it has multiple likely military applications [10]. For example, actigraphy using a wrist activity monitor has shown to be helpful in assessing and improving sleep in soldiers recently returned from deployment [11]. Scholars have focussed on assessing physical exertion and fatigue in military personnel by using non-invasive physiological monitoring devices [12]. Sensor systems to measure one or more biometric parameters for real-time prevention and monitoring of exertional heat illness in military personnel are very promising [13]. Research into the military use of PHM primarily engages with aspects like reliability, technical feasibility and the effect or outcome of PHM use.

However, developing and using PHM goes beyond its effects on health or the reduction of costs. There are also ethical considerations to take into account [14], such as privacy implications, the impact of PHM on the autonomy and safety of its user, the collection of data in relation to its intended use and increased or induced medicalisation of the user’s private or professional environment by the usage of PHM [15, 16]. If these ethical considerations are not adequately addressed, this may negatively affect the envisioned health benefits or healthcare cost reduction that PHM may bring, and may even be harmful [15, 17].

Soldiers adhere to military law and legislation and are part of a military culture, working and being deployed in extreme and often hostile environments. This raises the question if and to what extent the ethical considerations of PHM in the armed forces differ from those in civilian life. While there is research in which the ethical considerations of PHM in a civilian healthcare setting are addressed [17–19] and this is deemed important by some scholars [21], we did not find any published research specifically studying the ethical considerations of PHM in a military setting. Therefore, this study focusses on the *ethical dimension* of PHM within the armed forces. We define this *ethical dimension* as the implicit and explicit *normative descriptions* regarding the nature, qualities, risks and uses of PHM by users and other stakeholders, such as developers and policy advisors. *Normative descriptions* inform us, amongst others, about what is right and wrong, desired and undesired, responsible and irresponsible. These descriptions can be found within experiences, attitudes or (moral) questions, rules and agreements.

Knowledge about this ethical dimension can foster the responsible use of PHM in the armed forces and possibly other similar organisations working in high-risk environments, e.g. police and security. Responsible use concerns for example how to balance the interests between the need to accomplish mission success and to protect the soldiers’ privacy and personal life. The insights from this study can be used for the development, implementation and usage of PHM within the armed forces, thus harvesting all the benefits PHM potentially brings, taking disadvantages into account and protecting personnel against irresponsible use of PHM.

To examine the ethical dimension of PHM in the armed forces, we draw on an in-depth case study on an existing form of PHM, the Covid-19 Radar app, used in a national reserve unit within the Netherlands Armed Forces. In this case study, we aim to answer the following research questions: first, what values are expressed and/or deemed important by the users and other stakeholders of Radar regarding the development, implementation and usage of PHM? Secondly, which needs are expressed in relation to the support and handling of the identified ethical dimension of PHM?

Radar was developed by the Defence Health Organisation of the Netherlands Armed Forces, together with a civilian partner in technology, to gain insight in Covid-19-related military readiness and to support the reduction of the spread of SARS-Cov-2 within the armed forces. Radar, a mobile application, registered Covid-19-related symptoms by presenting a daily questionnaire (based on information from the National Institute for Public Health and the Environment of the Netherlands) to its users [20]. Submitting the questionnaire created personal advice telling a soldier to report for duty or to self-quarantine/isolate and report to a military physician. Besides this personal advice, Radar shed light on the development of Covid-19-related symptoms within military units, thereby supporting military physicians in their role as medical advisor to the unit commander. From November 2020 until April 2021, Radar was tested within a national reserve unit. The decision to test Radar in this unit was based on its willingness to participate in testing Radar without causing interference with regular military duties. The individual participation by soldiers was voluntary and anonymous. It constitutes one of the first uses of PHM within the Netherlands Armed Forces and is therefore a good case study to investigate the ethical dimensions of the use of PHM.

## Methods

### Study design

A qualitative, exploratory study was carried out using semi-structured interviews to allow users and other stakeholders to elaborate on which values and norms they deemed important, and which moral dilemmas and questions arose reflecting on their experience with and their role in Radar.

### Data collection

This case study focusses on a national military reserve unit consisting at the time of study of 143 persons, of which 78 participated in testing Radar. Reservists fulfil their military duties in addition to a job or study in civilian life. The entire unit was invited to participate in this study through a personal invitation. Other stakeholders were identified through purposive and snowball sampling and were personally invited to participate.

In total, twelve respondents – four users and eight stakeholders – participated in this study. Using a semi-structured approach, the users were interviewed in one dual and two individual interviews and the stakeholders in individual interviews. Due to Covid-19 restrictions, eight out of the total of eleven interviews were performed through online videocalls.

Questions in the semi-structured interviews included the following topics: motivation and thoughts about participation in Radar, reflections on the practical use and use of data of Radar, experienced or potential advantages and disadvantages, moral dilemmas regarding the use of Radar and respondents’ reactions to these dilemmas, and their need for support in dealing with moral dilemmas and questions.

### Data analysis

The interviews were audio-recorded, transcribed and thematically analysed using a systematic inductive approach [21]. First-order analysis focussed on respondent-centric codes. Through an iterative process of reviewing initial coding and the combining, clustering or collapsing of codes, second-order analysis led to the identification of various categories of themes. Each category represents a specific ethical dimension: value, norm and dilemma.

To support the analysis, the qualitative software program NVivo 12 was used to record codes and themes within the categories. Insights, discussions, thoughts and decisions were recorded in separate memos.

The first interview transcript was separately coded by all three researchers (DB, EvB, BM). A collaborative discussion followed this coding, which led to a tentative overview of several themes. In line with outcomes of the collaborative discussion, the remaining transcripts were coded by the primary researcher (DB). When in doubt about the most suitable coding, these transcript sections were discussed by the primary researcher (DB) with the other two researchers (EvB, BM) to reach consensus, eventually leading to a final coding set from which the second-order analysis started. During this second-order analysis we reflected on the emerging data and themes. During the thematic analysis we discovered three overarching categories reflecting the ethical dimensions of PHM in this case study. Changes in this stage of the analysis were discussed by the research group.

### Research ethics

Via a personal invitation letter, respondents were informed about the research objectives, data recording, data processing and confidentiality. In this letter, we emphasized the voluntary nature of participation. At the start of the interview this information was provided again. To ensure voluntary participation, all unit members were contacted personally and the commander of the unit was requested not to impose participation in this case study to his personnel. All participants provided written and oral informed consent to participate in the study. To ensure confidentiality, the interview data were anonymized and coded during handling, transport and storage.

The Medical Ethics Review Committee of VU University Medical Center confirmed that the Dutch Medical Research Involving Humans Act (WMO) did not apply. The need for approval was waived by the Medical Ethics Review Committee of VU University Medical Center. All experiments were performed in accordance with relevant guidelines and regulations. This study is registered at the Medical Ethics Review Committee of VU University Medical Center under the number 2021.0363.

All datasets used and analysed in relation to this study are available from the corresponding author on reasonable request.

## Results

In exploring the ethical dimension of PHM in the Netherlands Armed Forces, reflecting on the respondents’ role and experience with Radar, our findings show that several values and norms are deemed important and that some of them give rise to moral questions. In this section, we present our main findings.

**Fig. 1:**
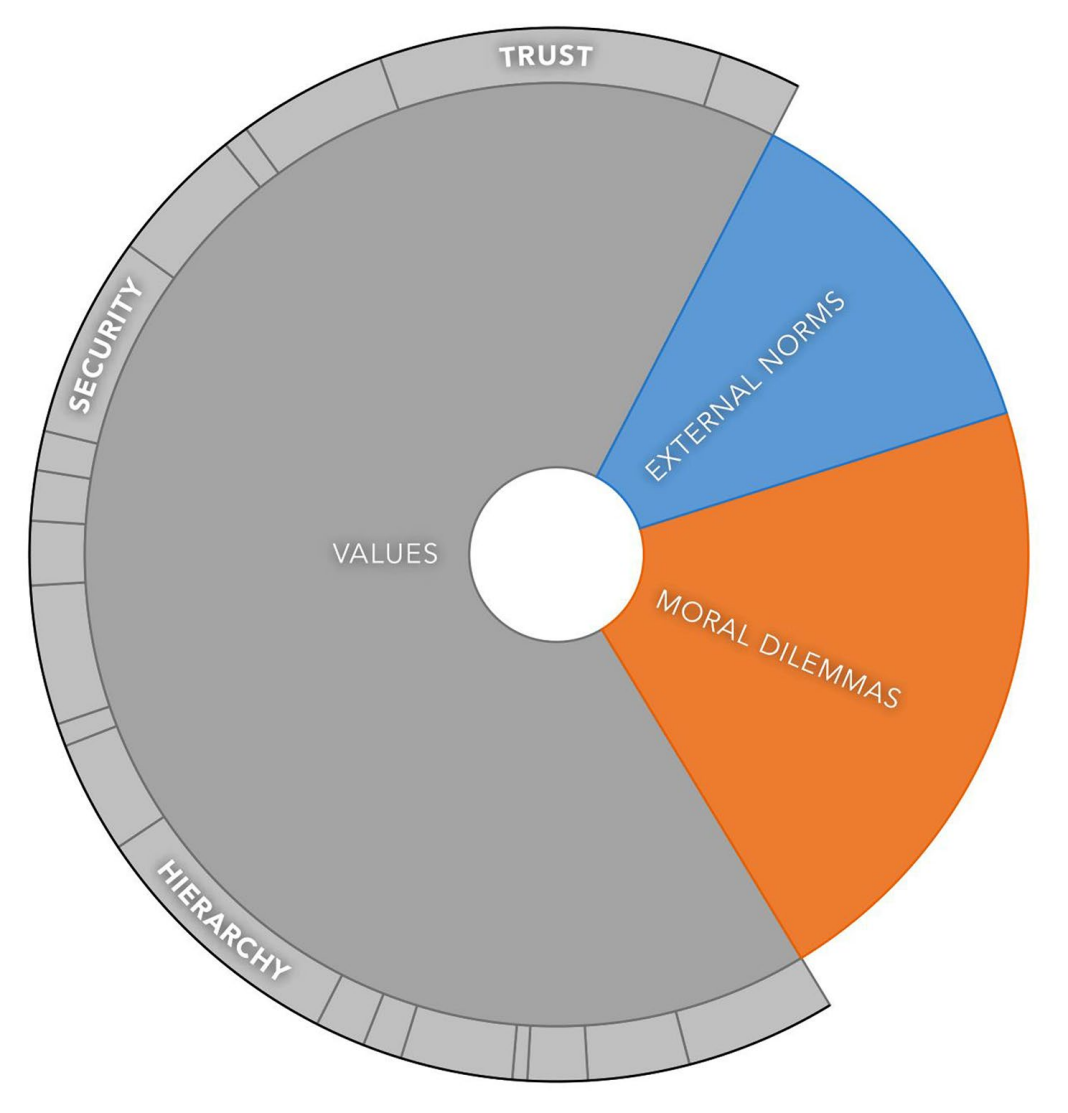
Overview of the three identified categories of ethical dimensions based on the data. The size of each section is defined by the amount of coding references per category

We formulated three interlinking categories reflecting the ethical dimensions of PHM in this case study, based on the data analysis and underlying research questions: (1) *values*, (2) *moral dilemmas* and (3) *external norms* (Fig. 1). *Values* were both explicitly and implicitly mentioned by respondents and are related to the organisation (i.e. the armed forces, commander and unit), PHM (i.e. primarily Radar itself) and to the respondents themselves. The category *moral dilemmas* is subdivided in experienced and presumed moral dilemmas and questions. *External norms* is the smallest category, measured by the number of statements made in regard to it. Examples of these norms, as stated by respondents, are using a legal basis to gather medical data from military personnel, the obligation for data minimalization (i.e. not gathering more data than strictly necessary for the intended purpose) and the need for transparent and careful handling of the obtained data. Because *external norms* are mainly derived from laws, both relating to the armed forces [22] as well as the General Data Protection Regulation [23], instead of based on respondents’ views, these norms and their underlying values are not further explored in this study. We will conclude this section with the expressed need for *ethics* s*upport*.

### Values

**Fig. 2:**
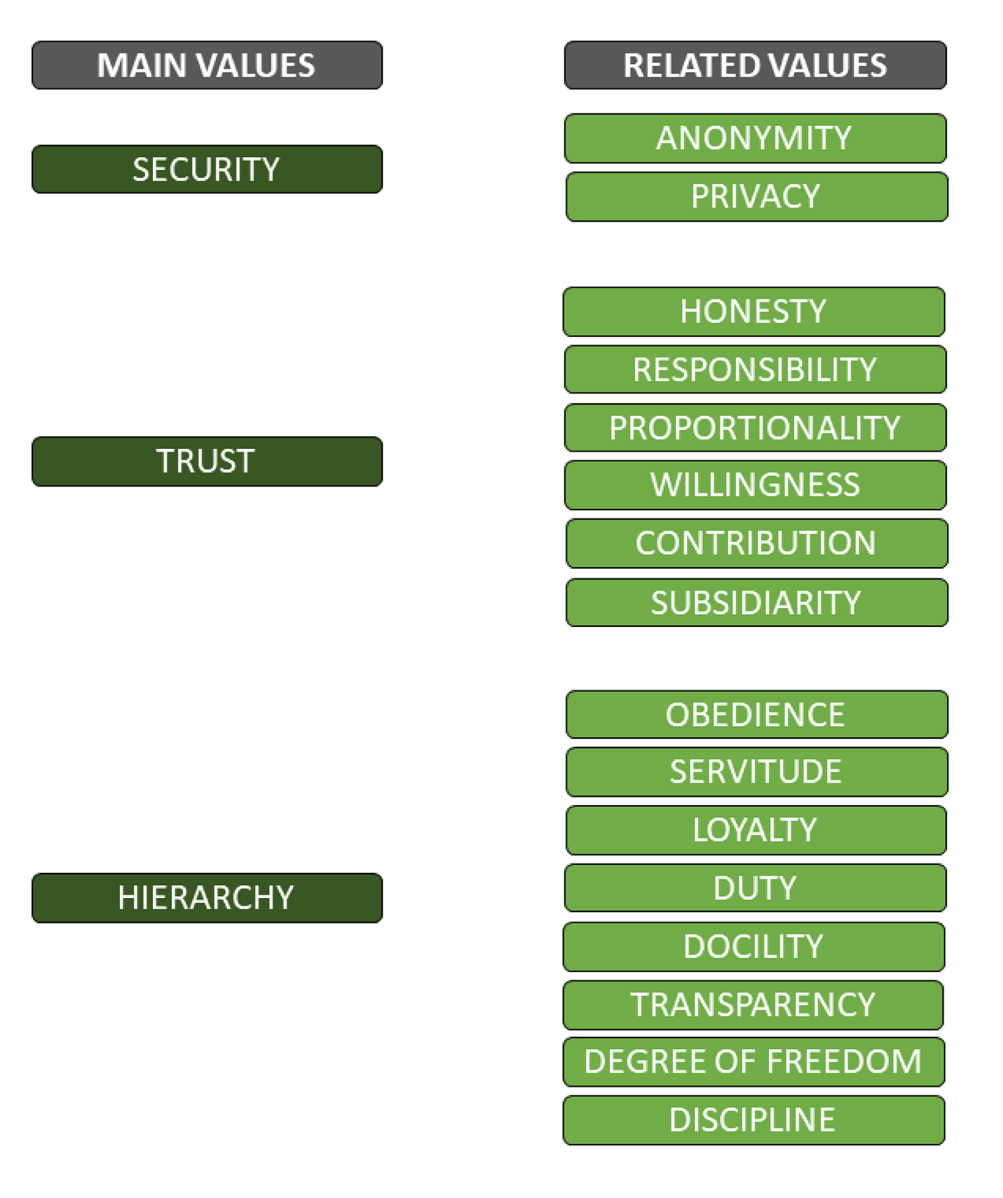
Overview of the identified main values and related values

The three values that stood out in the data are (1) *security (in relation to data)*, (2) *trust* and (3) *hierarchy.* Besides three *main values* we also identified several other values that are related to these main values. A preliminary overview of the main values and related values is presented in Fig. 2.

#### Values

Doubts were raised about what happens with the data gathered by Radar and to what extent this data poses risks for both the organisation – i.e. the armed forces – and for the individual users. This means *security* is an important value for the respondents. Stakeholders consider this value as being part of the armed forces organisation’s and the developers’ responsibility of protecting all parties involved (i.e. users, organisation, developers) against unauthorized and potentially harmful access to this data. These concerns lead both users and stakeholders to emphasize the importance of this value. As illustrated by two respondents, this value demonstrates a common understanding for both users and stakeholders about preserving security of data, thereby setting a norm.

*How about information security? Well, that’s closely related to the target group: soldiers. Because we don’t want data regarding soldiers to end up in the hands of foreign state actors and other rabble.* [stakeholder 6]

*And then I realised that there is an even greater risk: leaking this data. Through this, others and potentially malicious actors could be informed about the readiness of a unit.* [user 4]

#### Trust

The value *trust* is deemed key by all responding users in regard to participation in Radar. Trust is sculpted by and interacts with how the users experience their commander’s leadership, the military hierarchal organisation and the (potentially shared) goals of the organisation. It is also affected by the implications of Radar in regard to its potential outcomes and consequences and its effect on, for example, user privacy. The ability to trust and to what extent is moulded by the process of socialisation. According to the respondents, another factor playing an important role in the level of trust needed for respondents to participate in Radar is the context, i.e. the nature of the work, safety of the environment, and being a mission or peacekeeping activity.

To illustrate the role of leadership in regard to trust, a respondent expressed the following:

*If the commander tells me to go somewhere, I do that sort of blindly. Of course that is a matter of trust and so far that has never been compromised. So I continue. That makes my life a lot easier. That in fact you can trust the organisation, that little part of the organisation, blindly.* [user 3]

Trust in the military hierarchy, clear communication by superiors and a leader who makes participation – in this case in Radar – attractive affects users’ trust. This translates to how respondents (i.e. users) consider certain requests or orders.

*I temporarily donated my body and mind to the military. It is not a commercially free choice. For me that is really based on my confidence in the hierarchy. So, if people above me say something, I normally trust them, unless it is really crazy. Look, with this kind of thing* (i.e. participation in Radar), *I don’t have many considerations.* [user 3]

Several respondents value their trust in relation to privacy as soldiers differently than as civilians. As soldiers, they have a potential inclination to give up privacy more easily. This is considered necessary for the organisation to operate and, on the other hand, also seems related to the context in which soldiers operate.

*I understand completely that you have to give in on your privacy to provide clarity about your readiness.* [user 1]

*I find it easier to give up a bit on this* (privacy) *than I would in civilian life. Because, well, it’s just how our organisation works. Your information is needed to submit requests, gain access to material and that kind of things. So this* (i.e. participating in Radar) *was not a shockingly big step.* [user 2]

Trust is affected by the goals of the organisation and the relationships within units. As stated by some respondents, to serve the organisation and its goals, group interests often prevail over their own interests. Trust in this “higher” goal and in their colleagues seems to be the foundation for this prioritization. This norm, which seems common for soldiers, is, amongst other things, likely based on the process of secondary socialisation.

*When needed, the interest of the armed forces, in principle, prevails over my own interest.* [user 4]

*It’s like, putting a part of yourself aside to serve the higher goal of the team. I would do that. That would be different when asked for society or others. I find that much more difficult, because I think that some civilians create a mess in society and I don’t feel like cleaning that up. Within the armed forces there is much more camaraderie and the feeling that we are a unit.* [user 3]

Prioritizing the interests of the organisation above the user’s own interests shows a level of servitude typical for armed forces. The level of servitude depends on the context and degree of urgency though. A respondent mentions that during deployments he more easily puts aside his own interests, in this case privacy, than during regular peacekeeping activities.

Further building on the beforementioned higher goal of the organisation, there is another common ground for trust between users and the organisation, in relation to the security of (medical) data. Users express that it benefits both the organisation and themselves if their data is kept secure, thereby sharing the same goal.

*I trust that, again, it* (medical data) *stays within the organisation, because the organisation benefits from this in regard to military security.* [user 2]

A respondent reflects on his own personal background and how he was raised in relation to his high trust in science and the military organisation, thus positively influencing his motivation to participate in Radar. This reflection underpins the relevance of internalised values and norms through primary socialisation as an important building block of the ability to trust and subsequently, in this case, the willingness to participate in Radar.

*I think…I just went to university and did my PhD…so I know how science works. And that there is room for debate and people really overthink cases…That is always better than discussions with people who do not possess the necessary knowledge to judge something. Extending this view to the government, I trust the people who work there, whose job it is to make policy, more than some random other person. Besides, my mother has a uniformed profession, so I was taught just to listen to civil servants.* [user 4]

#### Hierarchy

This value, considered characteristic for a military organisation, plays an important role in participation in Radar. As explicitly stated by some respondents when talking about trust, in the organisation’s hierarchy and its leadership, it is implicitly connected to various other values, often considered typical for military organisations. These values are intertwined in the reactions of the respondents. Two of them being *obedience* and *docility*. Respondent users mention that, both related to Radar and in general, they obey a request or an order given by a superior without questioning.

*If something is asked by a military superior, often we simply comply.* [user 2]

This statement, amongst others, together with the users’ almost blind trust in their leader, is also linked to another value which resonates within military organisations: *loyalty*. According to some respondents, imposing something, such as rules or actions, is seen as a powerful and sometimes the only way to achieve a goal. It is a soldier’s *duty* to follow orders. Although Radar was tested on a voluntary basis, proposed organisation-wide deployment of Radar was only considered possible when mandatory. This obligation could be based on military legislation. Despite communicating typical values for military organisations like *hierarchy, obedience, docility, loyalty* and *duty*, respondents state that they assume that, even when the use of Radar is imposed, soldiers will not use Radar if they do not want to.

*Actually it is not up to the soldier* (i.e. deciding to use Radar). *To be clear, it is imposed* (i.e. in the proposed organisation-wide deployment). *Knowing that if a soldier really doesn’t want to, well, then he will not use it.* [stakeholder 6]

### Moral dilemmas and moral questions

No wide-shared moral dilemmas, either explicit or implicit, were found. In this section we make a distinction between (a) *experienced moral dilemmas and questions*, (b) *presumed moral dilemmas and questions*.

#### Experienced moral dilemmas and questions

According to respondents, there was tension between the level of diligence on the one hand, and timeliness on the other, during the development of Radar. To be and stay relevant, there was pressure for a quick launch of Radar. Following an insight in content-related concerns, such as the validity of the in-app questionnaires, a respondent states the following:

*I understand that we have to compromise in regard to the content of the app in order to keep momentum.* [stakeholder 7]

Respondents suggest closer and earlier involvement of experts in the process of developing and testing Radar. Although less explicit, their suggestion reflects a similar dilemma, one between diligence, accuracy and timeliness on the one hand, and about taking responsibility in regard to the content of Radar on the other.

*Looking at the content* (of Radar) *one might form an advisory group or expert group which could decide what should happen regarding the content.* [stakeholder 7]

This suggestion was a reflection of the respondent on what could be done differently in the future to improve the process of developing and testing PHM. Other suggested improvements for the future relate to the level of decision-making about organisation-wide deployment of Radar, in particular the timeliness and decisiveness.

*Timeliness and decisiveness should rapidly come into play. We are going to do it; we are not going to do it. It feels like this has been an extremely long process.* [stakeholder 3]

This illustrates a dilemma between making careful decisions – potentially without having all required information available – and reaching a decision on time. This dilemma is also related to unclarities and (moral) doubts about the governance organised around Radar, as shared by some respondents. Several stakeholders mention, as they describe it, an experienced lack in commitment on a higher level in the organisation regarding authorising the use of Radar and thereby taking responsibility. They also question who will give permission for the use of Radar and who decides what to do with the outcomes.

*How is the governance organised, who decides that you have to participate? And who decides what to do with the outcomes? Is that a commander, or a healthcare professional?* [stakeholder 7]

Another moral question revolves around the exclusion of civilian employees within the armed forces in regard to participation in Radar. This exclusion is based on the fact that civilians, in comparison to soldiers, cannot be obligated to use Radar. According to one respondent, this exclusion causes civilian employees to feel less appreciated. This may potentially damage an employee’s mental wellbeing and harm employer-employee relationships, consequently affecting employability.

#### Presumed moral dilemmas and questions

A presumed moral dilemma concerns the balance between individual and organisational interests, illustrated by the possible friction between the values of *privacy* and *security*. *Privacy* is related to the protection of the individual, while *security* relates to the protection of the organisation, according to a stakeholder. This individual interest of *privacy* needs to be weighed against the organisational interest of *security*, but these could collide. According to the same stakeholder, in regard to Radar, *privacy* and *security* were in line, but with other applications this might not be the case.

Some respondents express their willingness to compromise on their privacy (individual interest) in order to support the protection of their unit during a mission (organisational interest). During peacekeeping activities this *compromising* can be weighted differently.

*During deployment more is possible* (e.g. health monitoring) *than during peacekeeping activities. It’s a military organisation, so I don’t mind that much that this comes with certain expectations. But that should not go too far. Ultimately, personal interest outweighs organisational interest, during peacekeeping activities.* [user 2]

This insight leads to the moral question what a military organisation, considering the context (e.g. deployment or peacekeeping activity), might desire from its soldiers, knowing that soldiers are willing to compromise on certain individual interests to serve the organisation. This question is especially relevant when considering the aforementioned values like hierarchy, obedience and loyalty.

### Need for ethics support

In response to reflections on their actions and needs when confronted with experienced or presumed moral dilemmas or questions in regard to Radar, we asked stakeholders if and how any kind of ethics support could be of added value in addressing these difficulties.

One respondent suggests that a specialist in the field of ethics, who could initiate and lead a discussion to challenge a group of stakeholders to think further, could be helpful to identify or address potential moral dilemmas and questions. Another respondent proposes the formation of a group composed of different disciplines, such as users, commander, health specialist, legal counsel and free-thinker, to analyse the needs and possibilities in regard to future health monitoring projects, based on different scenarios.

*I have the need to, in fact, think from scenarios. Together with subject matter experts…to develop scenarios and then look at the frame of reference* (i.e. what do we find acceptable in what situation?). [stakeholder 2]

Although not clearly expressed by most respondents, these suggestions show that there are some needs, indicated by respondents, regarding potential ethics support for dealing with moral dilemmas and questions related to PHM.

## Discussion

The purpose of this study is to explore the ethical dimension of PHM in the armed forces in order to foster a responsible use of PHM within the military. We propose values, moral dilemmas and external norms as three relevant ethical dimensions of PHM. Trust, primarily related to leadership, appears to be a key value, followed by hierarchy and security. These values appear related to multiple other identified values, including willingness, loyalty and privacy. No widely shared moral dilemmas, either explicit or implicit, were found. The few moral dilemmas that were found are mostly attributed to the content of Radar, unclarities about the governance around implementing and using Radar, and the influence of the context on the users’ willingness to serve organisational interests. Despite these ethical dimensions, in general the stakeholders did not report a strong need for ethics support. Some stakeholders mentioned the potential role of an ethicist or a multidisciplinary advisory group. In this section, we use the three main values identified as a point of departure to discuss the relevance and potential implications of our findings for the future use of PHM within the armed forces.

Security of data is highlighted by users as well as stakeholders. Multiple scholars recognise the security of wearable technology, including PHM, as an ethical issue [24, 25]. Unauthorised access could potentially lead to personal threats to users, thereby outweighing the initial benefit of using the technology. Furthermore, within the armed forces, data is often considered a strategic asset [26]. PHM data is valuable, yet therefore also strategically vulnerable intelligence, as it holds information about military readiness. For this reason, data breaches could pose a substantial threat to armed forces. Protection of sensitive information is therefore beneficial for both users and the military organisation, necessitating a substantial degree of data protection. This means an analysis assessing not only the impact of data protection for users of PHM (e.g. Data Protection Impact Assessment) is necessary, but also the impact of data protection for the military organisation. Consequently, the advantages of PHM should be weighed against its disadvantages when data is obtained by unauthorised parties, potentially compromising the safety of PHM users or the strategic advantage of the use of PHM. In our study, both users and stakeholders agreed on the importance of security. If this is not the case, and if the implications of a mismatch are not recognised and discussed, this could lead to the use of PHM in which organisational interests prevail over individual interests without being debated. This potential dilemma makes analysing the security of data, from both a user and organisation point of view, part of assessing the morally responsible use of PHM.

Security of data goes beyond the protection of digital data against unauthorized access, corruption or theft. It appears to be closely related to the value trust, expressed as the confidence that users have that the military organisation will do its utmost to protect the data provided by soldiers. To contribute to trust in PHM, systems must allow users to review and control their data [27]. To some extent this was also the case with Radar, which contributed to trust in this particular system. With future PHM applications on the horizon, or applications in a different context (e.g. deployment), the relevant trust issues might be different. Even in a military organisation, where participation in PHM could be made mandatory and where personal health data could serve only a military and not solely a personal goal, non-use or manipulation of the PHM by users, when the level of trust is low, can have serious consequences. For example, operational decisions could be based on the wrong data, thus affecting soldiers and the mission. Options for users to control their data in a transparent manner can be an effective way to promote trust in PHM systems and contribute to the compliance and trustworthiness of PHM. Furthermore, in light of the ongoing ethical debate about security, privacy, data ownership [28] and data increasingly being seen as a human right [29], armed forces should closely consider who actually owns a soldiers PHM data, and who can access it and under what circumstances.

Our findings show that users have a high degree of trust in their commander, the organisation and its intentions, resulting in a high willingness to participate in PHM via Radar. This is in line with the cross-disciplinary definition of *trust* set by Rousseau, Camerer and Sitkin: *Trust is a psychological state comprising the intention to accept vulnerability based upon positive expectations of the intentions or behaviour of another* [30]. The military context, especially during deployments, often poses a high-risk environment where vulnerability, uncertainty and interdependence are unmistakable situational antecedents of trust. This context creates a need to trust (i.e. to accept these antecedents) for soldiers to operate and to, sometimes literally, survive in high-risk environments. They must assume that their commander and their colleagues are competent and honourable and that their intentions are benevolent [31]. It makes trust an important core value, also in regard to PHM, where the same assumptions might apply. Our findings indicate that trust is influenced by both (inter)person and organisation-based factors. Trust can be seen as a primary need in a military organisation [32], but there is a potential downside to this trust expressed by the users. It shows a possible (latent) vulnerability of military users of PHM regarding their ability to balance their risks and benefits. An example of this vulnerability is shown by the absence of major privacy concerns amongst the respondent users, whereas it is precisely these privacy concerns that are a principal ethical issue in the general literature about PHM [33, 34]. The reserve unit in this study was not deployed to combat Covid-19. The users were not in a high-risk environment such as a combat mission, a context which may even require a higher level of trust. The users nonetheless did not specifically weigh their personal risks and benefits in regard to participating in Radar; organisational interests were deemed more important. Respondents based their decision to participate on their primary trust in their commander and the organisation as a whole. Richards and Hartzog state that “trust allows us to develop long-term, sustainable information relationships by sharing meaningful but often sensitive information and having sincere exchanges, with the confidence that what we share will be used for our benefit and not come back to haunt or harm us” ([35], p. 1213). With this in mind, and contemplating the absence of privacy concerns in this case study, it is likely that the respondent users trusted that their data would be used for their and the organisation’s benefit, without causing them harm, now and in the future. Trust as a core value may explain the absence of experienced moral dilemmas among the respondent users, as they did not question their commander and organisation. They might be less critical as this great trust may imply there is no need to be critical.

We found no widely shared moral dilemmas experienced by our respondents, which is remarkable in a time when health monitoring by employers can be seen as controversial, especially when technologies find their way from the professional workplace to an individual’s private domain [36]. Nevertheless, it seems most respondents experienced no moral doubt; they thought that monitoring Covid-19-related symptoms was necessary to assess military readiness in times of uncertainty regarding the course and impact of the Covid-19 pandemic and the related readiness of the armed forces. A high level of trust in their leader, in combination with a sense of urgency to use Radar, further supports this absence of dilemmas. Presumably this great trust, paradoxically, may also conceal parts of the ethical dimension of PHM. Together with the limited experienced and presumed moral dilemmas and questions, it comes as no surprise that no strong need for ethic support was expressed. We argue that a moral responsible use of PHM, in the light of this paradox, needs ethics support to uncover and address these potentially hidden ethical aspects.

Hierarchy, another key value for military organisations, is also related to trust. Based on the reactions from the users, the foundation for this hierarchy to work – i.e. a command or request is followed – is based on trust in their leader and the organisation. While not expressed by the respondents, hierarchy, like trust, also creates a certain vulnerability for service members, as they are expected to follow commands and often are not in the position to make decisions on their own. Although this hierarchy serves a specific goal, namely a clear command and control structure, we think the vulnerability that is hidden in this hierarchy needs to be taken into account to ensure morally responsible use of PHM.

Previous research has shown that if potential users trust the PHM system, they are more likely to use it [37]. Our research builds on this by providing an indication that trust in their leaders and the organisation, as opposed to trust solely in a particular PHM system, is a potentially strong determinant of acceptance and use of PHM in the armed forces. Trust and hierarchy, which are pervasive military values, also bring a vulnerability in relation to PHM in a low-risk environment such as peacekeeping activities. This vulnerability lies, amongst others, in the question to what extent military users are able to balance the risks and benefits of voluntary as well as mandatory actions or interventions, such as PHM, to protect their personal interests and still support organisational interests outside of high-risk environments. This vulnerability also encompasses the principle of informed consent, which is a prerequisite for any medical or research procedure, based on the value of autonomy [38]. Several scholars question whether true informed consent is even possible in the armed forces due to the diminished autonomy of soldiers caused by power relations, military command structures, training and processes of socialisation. These aspects are all aimed at subsuming one’s individual desires to the needs of the greater cause [39, 40]. The expressed trust by the respondent users, the possible negative effect this has on their autonomy and the potentially concealed moral dilemmas align with this doubt about the possibility of true informed consent in the armed forces.

Although there is likely some overlap, it is unclear to what extent our findings are applicable to systems other than active monitoring systems – i.e. where the users actively have to answer a daily questionnaire – like Radar. On the one hand, values like security, trust and hierarchy can play an even more crucial role in passive monitoring systems like wearables, since users have less influence and control over the data they share. On the other hand, passive monitoring implies that users are not constantly made aware about the fact that they are monitored, which could raise less attention to a value like trust.

In a military organisation, hierarchy and the functional and necessary trust in leaders, orders and the organisation continue beyond the military context of high-risk environments. This brings along a moral responsibility to conscientiously handle trust and hierarchy, in particular outside high-risk environments, and to build in safeguards to protect this potentially vulnerable military population [40–42]. This responsibility includes carefully considering the added value of ethics support when dealing with PHM systems, especially in times of large-scale data misuse and theft [43], and continuous threats to privacy. Although not substantively reported and discussed in this article, external norms like laws, rules and regulations, play an important and indispensable role in ensuring safe, accurate and ‘privacy-correct’ use of PHM [44]. In the European Union, the General Data Protection Regulation, the Medical Device Regulation, as well as national law and specific military law and regulations need to be designed in such a way that they protect users against unsafe, inaccurate and privacy-undermining PHM.

This study can foster awareness of the ethical dimensions of PHM and help to identify and address key ethical dilemmas in future PHM systems. Furthermore, these insights complement existing debates in the field of military medical ethics on examining the ethical dimension of using technical medical knowledge for military purposes [45].

### Strengths and limitations

The major strength of this study is that, by our knowledge, this is one of the first studies researching the values, norms, moral dilemmas and questions of PHM in the armed forces. It does so by harvesting insights from empirically gathered data from multiple groups, i.e. users and different stakeholders, based on their experiences, instead of a top-down expert approach on the subject. It complements the existing literature on the ethical considerations of PHM in civilian (public) healthcare, and also adds to the domain of military medical ethics. The results can be used for future research, guide designing normative frameworks or even be utilized by policymakers.

There are also some limitations to take into account. The findings at this national reserve unit are potentially not representative for the rest of the armed forces, because this unit has different demographics, such as a higher median age and higher level of education. Also, their contribution to testing Radar was based on their willingness to participate. This, together with the voluntary nature of participation, possibly created a positive attitude towards PHM to start with, thereby influencing the results of this study. Furthermore, there was a low response rate from the users. Both might affect the composition and hierarchy of values found. The preliminary overview of values presented (Fig. 2) is not a systemic arrangement of values. Their relation to one another and the implications of these values for the responsible use of PHM need to be further studied. The low response rate could be due to the high turnover of personnel in this unit. Alternative explanations can be the infrequent use of work email, so that the users were not able to respond to the invitation to participate on time; not being able to participate due to their regular, non-military work; or just not feeling the urgency to participate. Due to privacy regulations, we were not able to recruit participants through other communication channels than their work email. Furthermore, members of the unit who did not use Radar did not participate in this study. As a result, we potentially missed out on relevant normative descriptions regarding the ethical dimension of PHM.

Lastly, we interviewed the participants a few months after they had stopped using Radar, when the Covid-19 situation was changing rapidly (e.g. fluctuating disease incidence, start of national vaccination campaign). This might have influenced the respondents’ memories, their views on what values were important to them, and the moral dilemmas deemed important by the respondents while they were still using Radar. To compensate for this potential recall bias, we presented a short overview of the current Covid-19 situation compared with the situation when Radar was used at the start of each interview, in order to refresh the respondents’ memory.

## Conclusion

This study explored the experiences and viewpoints of users and other stakeholders with respect to the ethical dimension of PHM in the Netherlands Armed Forces. The findings of this study shed light on the different values at play according to various stakeholders. It also shows that respondents, in general, did not experience many or strong moral dilemmas or questions. Despite this, some suggestions for ethics support were identified. This study highlights security of data, trust and hierarchy as important values. Trust in regard to PHM is related to leadership, (shared) goals, the implications of PHM and processes of socialisation. These values also cause a certain vulnerability to military users when personal and organisational interests are not aligned. Trust potentially hinders a clear deliberation about the ethical dimensions of PHM. The context in which PHM is developed and used plays an important role in acceptance and compliance.

The findings of this study elucidate various ethical dimensions of PHM and its use within the Netherlands Armed Forces. They highlight the moral responsibility for the armed forces to devote attention to the ethical dimensions of PHM. Future ethics support can assist in taking up this responsibility by uncovering and addressing potentially hidden moral dilemmas. Suggestions for future research include exploring further the vulnerabilities of service members, given the identified values of trust and hierarchy, and uncovering hidden moral dilemmas regarding the development, implementation and use of PHM, as well as exploring various ways of ethics support in order to contribute to the responsible use of PMH in the armed forces. Differentiation between active and passive monitoring systems, as well as variation in the context – e.g. active military duty or reserve unit, peacekeeping activities or deployment, prevention or treatment of illness – could be valuable elements in uncovering more values and moral dilemmas at play.

## Data Availability

The datasets used and/or analysed during the current study are available from the corresponding author on reasonable request.

## List of abbreviations

PHM: personal health monitoring
